# Systematic review of rheumatic disease phenotypes and outcomes in the Indigenous populations of Canada, the USA, Australia and New Zealand

**DOI:** 10.1007/s00296-016-3623-z

**Published:** 2016-12-17

**Authors:** Kelle Hurd, Cheryl Barnabe

**Affiliations:** Cumming School of Medicine, University of Calgary, 3330 Hospital Dr NW, Calgary, AB T2N 4N1 Canada

**Keywords:** Indigenous, Rheumatic disease, Disease activity measures, Patient-reported outcomes

## Abstract

We performed a systematic review designed to characterize clinical phenotypes and outcomes in Indigenous populations with rheumatic disease to enhance the understanding of how rheumatic disease presents in Indigenous populations and allow for better projection of the healthcare needs of the communities affected. A systematic search was performed in medical (Medline, EMBASE, CINAHL), Indigenous and conference abstract databases (to June 2015). Search terms for Indigenous populations were combined with terms for inflammatory arthritis conditions, connective tissue disorders, crystal arthritis and osteoarthritis. Studies were included if they reported on disease features, disease activity measures, or patient-reported outcomes in Canadian, American, Australian or New Zealand Indigenous populations. Data were extracted in duplicate, and a narrative summary was prepared. A total of 5269 titles and abstracts were reviewed, of which 504 underwent full-text review and 85 met inclusion criteria. Nearly all the studies described outcomes in the North American populations (*n* = 77), with only four studies from Australia and four studies from New Zealand. The majority of studies were in rheumatoid arthritis (*n* = 31) and systemic lupus erythematosus (*n* = 19). Indigenous patients with rheumatoid arthritis had higher disease activity and reported more significant impact on patient-reported outcomes and quality of life than non-Indigenous patients. Spondyloarthropathy features were described in North American populations, with most patients having advanced manifestations. In systemic lupus erythematosus, nephritis was more frequent in Indigenous populations. Gout and osteoarthritis were more severe in New Zealand Maori populations. The existing literature supports differences in disease phenotype and severity in Indigenous populations of Canada, America, Australia and New Zealand. We encourage investigators in this area of research to undertake contemporary studies that disentangle differences between phenotype and severity that are biologic in etiology or merely reflecting differences in access to care and that provide a longitudinal assessment of outcomes in more diverse populations.

## Introduction

The study of rheumatic disease prevalence in Indigenous populations of North America, which include Canadian populations of First Nations, Métis and Inuit people (collectively referred to as Aboriginal Peoples) and American populations of American Indian/Native and Alaska Natives, highlights increased prevalence rates of osteoarthritis, inflammatory arthritis and connective tissue disease conditions, influenced by tribal ancestry in the First Peoples of the continent [1–3]. It has been proposed that important phenotypic differences also exist between Indigenous and non-Indigenous populations with rheumatic diseases. For example, an Aboriginal cohort with rheumatoid arthritis followed at a tertiary care center in Manitoba were more frequently seropositive and had worse HAQ scores than a Caucasian group [4]. In First Nations, American Indian and Alaska Native populations with rheumatoid arthritis, more extra-articular manifestations, erosive disease and more severe radiographic findings in Indigenous patients are described [1, 2]. In systemic lupus erythematosus, First Nations people in Manitoba had higher disease activity scores at diagnosis, with more frequent vasculitis, proteinuria and cellular casts, and worse damage scores over the disease course [5]. Of note, Australia and New Zealand’s Indigenous populations, the Australian Aborigines and New Zealand Maori, respectively, have not been included in any of the prior reviews, but share commonalities with the North American Indigenous populations. Canada (until 2016), the USA, Australia and New Zealand are the only countries that rejected the United Nations Declaration on the Rights of Indigenous Peoples [6], and share similarities in difficulties in access to healthcare coverage [7], which may influence clinical outcomes.

These clinical outcomes, and whether phenotypic differences truly exist between Indigenous and non-Indigenous populations, are important issues to explore further to inform clinical practice and health systems design. Biologic reasons are proposed [2], which may inform individual treatment recommendations, but unwarranted variations in access to adequate healthcare resources may also affect disease outcomes and would need to be addressed by health policy and health service delivery changes. We thus performed a systematic review designed to characterize clinical phenotypes and outcomes in Indigenous populations, while also identifying studies where a comparison to non-Indigenous patients was made, which will provide improved understanding of how rheumatic disease is present in Indigenous populations and allows for better projection of the healthcare needs of the communities affected.

## Methods

### Data sources

We performed a broad search using medical literature databases and Indigenous specific online indexes and organization websites identified with the help of a medical librarian. Medical literature databases searched included Medline (1946–June 2015), EMBASE (1980–June 2015) and CINAHL (1996–June 2015). Indigenous specific online indexes and organization websites searched (June 2015) were the Circumpolar Health Database, Health Info Net, Metis Health Database, Native Health Database, Native Indigenous Studies Portal and The First Nations Periodical Index. We also did a search of each country’s government websites for relevant publications. References of relevant identified studies were reviewed for additional primary references.

### Search terms

This study was part of a larger review to characterize the epidemiology, clinical outcomes and healthcare service utilization of arthritis conditions for Indigenous populations of Australia, Canada, New Zealand and the USA. Keywords and Medical Subject Headings (MESH) for the terms ‘arthritis’ and ‘indigenous populations’ were selected with the assistance of a medical librarian. An example of the search strategy (conducted in Medline) is provided in ‘Appendix.’ We used an expanded version of a ‘3E’ search strategy developed for identifying studies on inflammatory arthritis [8], including validated terms that have been used to identify other arthritis conditions such as osteoarthritis [9], gout [10], juvenile idiopathic arthritis, systemic lupus erythematosus [11], scleroderma, polymyositis, dermatomyositis, Sjögren’s syndrome, as well as terms for general arthritis and rheumatic disease. Search terms for Indigenous populations of interest used both global and local terminology. Only the arthritis search terms were used during the Indigenous online indexes and websites review. No language or publication date restrictions were imposed during the electronic search. The literature search was not limited by specific clinical outcomes terms.

### Inclusion criteria

We identified cohort, case–control and cross-sectional studies specifying estimates of clinical outcome measures in Indigenous populations [12] from Australia, Canada, New Zealand and the USA with arthritis conditions (based on the Public Health Agency of Canada definition [13]) of osteoarthritis, rheumatoid arthritis, juvenile idiopathic arthritis, ankylosing spondylitis, psoriatic arthritis, Reiter’s disease, other spondyloarthropathies, systemic lupus erythematosus, scleroderma, polymyositis, dermatomyositis, Sjögren’s syndrome or gout. Studies were included if they reported on one of the standard measures of outcomes, including, but not limited to those suggested by OMERACT (Outcome Measures in Rheumatology) for pharmacologic and complementary interventions. Clinical characteristics or features include: tender joint counts, swollen joint counts, patient global, inflammatory markers [erythrocyte sedimentation rate (ESR), C-reactive protein (CRP)], composite disease activity scores, Health Assessment Questionnaire (HAQ), morning stiffness duration, radiographic imaging (either as % with erosions or actual score of damage), Quality of life (any validated scale), serology results [rheumatoid factor (RF), anti-cyclic citrullinated peptide (anti-CCP), anti-nuclear antibody (ANA)], extra-articular features (nodules, sicca complex, vasculitis, interstitial lung disease, neuropathy, etc.), patient-reported outcome measures including visual analogue scales for pain or fatigue.

### Data collection and analysis

Two review authors (CB, KE) independently screened titles and abstracts and performed the full-text review. Disagreements were resolved by consensus. Review articles and articles with secondary data were included during the initial eligibility screening and when available, the primary studies were obtained and used for data extraction. The authors then extracted the data from included studies using standardized and pretested data extraction forms to collect information on country of study, Indigenous population and number, comparison population and number if relevant, study design, type of arthritis and outcomes.

### Data synthesis

A narrative synthesis was prepared given the heterogeneity of populations, study methods and outcomes reported.

## Results

### Study characteristics

**Fig. 1:**
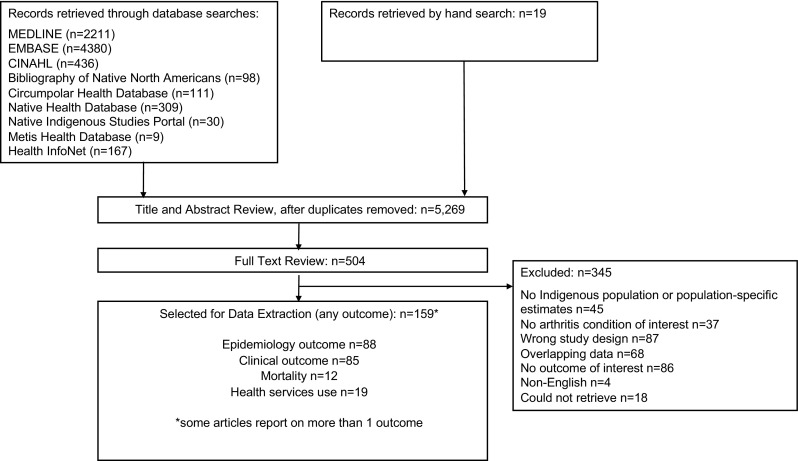
Article Identification and Selection

This study was part of a larger initiative to synthesize epidemiology, clinical outcomes, mortality and health services use in Indigenous populations in the four countries of interest. A total of 5269 titles and abstracts were reviewed, of which 504 underwent full-text review for any of the three outcomes above, and 85 were included for extraction of clinical outcomes (Fig. 1). Nearly all the studies described outcomes in the North American populations (*n* = 38 American, *n* = 37 Canadian, plus *n* = 2 studies reporting both American and Canadian data), with only 4 studies from Australia and 4 studies from New Zealand. The majority of studies were in cohorts of patients with rheumatoid arthritis (*n* = 31) and systemic lupus erythematosus (*n* = 19), with studies in all rheumatic diseases (osteoarthritis *n* = 7, spondyloarthritis conditions *n* = 13, inflammatory arthritis not meeting criteria for a specific type *n* = 7, self-reported arthritis *n* = 2, scleroderma *n* = 4, Sjogren’s syndrome *n* = 1, gout *n* = 1, juvenile idiopathic arthritis *n* = 9, juvenile SLE *n* = 7, juvenile spondyloarthritis *n* = 3) except polymyositis and dermatomyositis identified.

### Self-reported arthritis

Both identified studies utilized a cross-sectional design. Ferucci’s study recruited 9968 Southwest American Indian and Alaskan Native participants [14]. Those with arthritis, relative to those without arthritis, had worse SF-12 Physical Composite Scores (Alaska mean 43.9 vs 52.6; American Indian mean 42.7 vs 49.7; both *p* < 0.001) but not SF-12 Mental Health Composite Scores. In both locations, approximately 50% of the subjects with arthritis reported pain interfering with work, compared to 15–20% of those without arthritis (adjusted OR 3.4 and 3.1, respectively). In Lawrence’s analysis of the National Health Interview Survey (1989–1991), 22.6% of American Indian and Alaska Natives with arthritis reported activity limitations, compared to 17.6% of Whites, 24.5% of Blacks, 13.0% of Asian and Pacific Islanders [15]. Thus, in the American Indigenous populations self-reporting arthritis diagnoses, significant limitations in physical function are apparent and in excess to that seen in non-Indigenous populations.

### Osteoarthritis

Seven studies describe disease features and impact in Indigenous populations with osteoarthritis [16–22]. Osteoarthritis was the forth leading cause of years lost to disability in American Indians [19]. The other studies in American Indians (1) report the frequency of a positive ANA in osteoarthritis being 63% in a cross-sectional study design, and in unknown titer [17]; (2) describe in a cross-sectional study swollen (mean 1.4, SD 2.0) and tender (mean 4.4, SD 4.1) joint counts, physical function (mean Health Assessment Questionnaire score 0.46) and pain (mean 5.6, SD 2.5) [16]; and (3) describe radiographic findings in a cohort study design using the Kellgren/Lawrence scoring system, along with individual-radiographic-feature scoring systems of qualitative and quantitative assessments [18]. A single study in Canadian Inuit described that all patients with osteoarthritis had mild impairment in function, frequently had mild–moderate disease activity, nearly universal confirmation of degenerative changes on radiographs and rare ANA positivity with no patients having a positive rheumatoid factor [20]. A descriptive study of an Australian Aborigine cohort characterized clinical and radiographic findings concluding that significant degenerative arthritis was uncommon, but with the majority of osteoarthritis affecting weight-bearing joints and the lumbar spine [22]. A longitudinal study of Maori and non-Maori populations undergoing hip and knee arthroplasty compared pre- and postoperative scores relative to osteoarthritis, including the Oxford and WOMAC scores, and SF-12 General Health (PH) and Mental Health scores between 2005 and 2009 [21]. Preoperative disease specific function was significantly worse in Maori (mean Oxford scores 10.10 vs 11.26 and WOMAC 76.24 vs 73.54). Although both Maori and non-Maori improved postoperatively, there were smaller overall improvements in Maori. SF-12 PH scores were similar between groups, but SF-12 MH scores were worse in Maori preoperatively, and again at 1- and 5-year time points. These results suggest variation in osteoarthritis manifestations across Indigenous populations, although with limited homogeneity in the clinical aspects studied and reported. The single study looking at the outcomes of surgical intervention highlights higher disease severity at time of procedure, thus perhaps contributing to the limited improvement gained through the surgery.

### Rheumatoid arthritis

There were 31 studies identified [4, 17, 20, 23–50], with nine that included a comparison of clinical features to a control population, mostly Caucasian, but also to some control populations characterized as non-Indigenous (Table 1).

**Table 1:** Studies on clinical features and outcomes in rheumatoid arthritis in Canadian, American, Australian and New Zealand Indigenous Populations

References	Indigenous population	RA classification criteria	Total number of Indigenous participants *N* or M:F if available	Control population	Total number of control *N* or M:F if available	Features reported
*Studies with comparison group*						
Jacono [25]	Canadian Aboriginal	ARA (Ritchie)	5:38	Caucasian	53:89	Demographics, joint distribution, severity (surgical requirements)
Hitchon [26]	Canadian First Nations	NR; includes some patients not meeting criteria	143	Caucasian	409	Demographics, patient-reported outcomes
Poole [27]	American Indian	ACR 1988	0:17	Caucasian	0:15	Demographics, patient-reported outcomes
Genovese [28]	American Indian	NR	62	Caucasian	81	Demographics, disease activity, joint counts, inflammatory markers, patient-reported outcomes, serology
Peschken [4]	Canadian Aboriginal	ACR 1987	101:380	Caucasian	289:1026	Demographics, joint distribution, disease activity, joint counts, inflammatory markers, patient-reported outcomes, serology
Hitchon [24]	Canadian First Nations	NR	NR	Non-First Nations	NR	Demographics
O’Neil [29]	Canadian First Nations	NR	22:128	Caucasian	32:122	Demographics, joint counts, inflammatory markers, patient-reported outcomes, radiographic findings, serology, severity
Barnabe [30]	Canadian Aboriginal	NR	90	Non-Aboriginal	1400	Demographics, disease activity, joint counts, inflammatory markers, patient-reported outcomes, serology, quality of life measures
Hitchon [24]	Canadian First Nations	NR	NR	Non-First Nations	NR	Demographics, mortality
*Descriptive studies*						
Templin [31]	American Indian	ACR 1987	8:29	NR	NR	Demographics, disease features, joint counts, patient-reported outcomes, radiographic findings, serology, severity
Burch [32]	American Indian	NR	42	NR	NR	Serology
Gofton [33]	Canadian Aboriginal	ARA 1958	10:4	NR	NR	Patient-reported outcomes, radiographic findings, serology
Beasley [34]	American Indian	NR	17	NR	NR	Demographics, disease features, radiographic findings, serology
Willkens [35]	American Indian	ARA and NY Criteria	0:36	NR	NR	Radiographic findings, serology
Harvey [36]	American Indian	Bennett and Wood	4:10	NR	NR	Serology
Harvey [37]	American Indian	ARA 1958	3:9	NR	NR	Demographics, disease features, radiographic findings, serology
Oen [20]	Canadian Inuit	NR	6	NR	NR	Demographics, radiographic findings, serology, severity
Boyer [38]	Alaska Native	ARA 1958	14:33	NR	NR	Demographics, patient-reported outcomes, radiographic findings, serology
Jacobssen [39]	American Indian	Rome 1961	20:65	NR	NR	Joint distribution, disease features, serology
Scofield [40]	American Indian	ACR	4:41	NR	NR	Demographics, disease features, serology
Hirsch [41]	American Indian	ACR and Rome	88	NR	NR	Radiographic findings
Atkins [42]	Canadian First Nations	ARA	23	NR	NR	Demographics, disease features, serology
Coutts [43]	Canadian First Nations	ARA 1987	3:14	NR	NR	Demographics, serology
Poole [44]	American Indian	ACR 1988	0:4	NR	NR	Demographics, patient-reported outcomes
El-Gabalawy [45]	Canadian First Nations	ACR	53:213	NR	NR	Demographics, serology
El-Gabalawy [46]	Canadian First Nations	NR	8:74	NR	NR	Demographics, serology
Poole [47]	American Indian	NR	29	NR	NR	Demographics, patient-reported outcomes
El-Gabalawy [48]	Canadian First Nations	ACR 1987	14:91	NR	NR	Demographics, inflammatory markers, serology
Gaddy [17]	American Indian	ACR	40	NR	NR	Demographics, serology
Ferucci [49]	Canadian First Nations and Alaska Native	ACR 1987	11:71	NR	NR	Demographics, serology
Barnabe [50]	Canadian First Nations	NR	19	NR	NR	Serology

*ARA* American Rheumatism Association, *NR* not reported, *ACR* American College of Rheumatology; *NY* New York

#### Rheumatoid arthritis disease characteristics and disease activity

In comparison studies, most authors reported that Indigenous patients were younger at disease onset [4, 24, 25], as much as 9–14 years younger. The frequency of nodules was between 4 and 46% when reported [31, 34, 37, 39, 42] and associated Sjogren’s syndrome or sicca symptoms between 15 and 27% [31, 37, 42]. The study of the Nuu-Chah-Nulth tribe was the only publication describing the frequency of other extra-articular manifestations such as lung disease and vasculitis [42], and in a study of the Alberta Aboriginal population, the frequency of comorbidities was described, increased compared to the non-Aboriginal group [30]. Focusing on disease activity measures, two of the comparison studies report average DAS28 scores, with Oklahoma American Indians having a modest but nonsignificant trend to higher scores compared to Caucasian controls [28], whereas Alberta Aboriginal patients had higher DAS28 scores at initiation of biologic treatment compared to non-Aboriginal patients (6.11 vs 5.19, *p* < 0.001) [30]. In all comparison studies reporting tender or swollen joint counts, there were no significant differences between Indigenous groups and their comparison cohorts [4, 28, 29]; however, the publication contrasting Alberta Aboriginal to non-Aboriginal population demonstrated slower rates of improvement in these counts over time in the Indigenous group during biologic treatment [30]. Manitoba First Nations patients were less likely to achieve remission compared to Caucasian patients (20 vs 58%) [26]. Physician evaluation of global disease activity was significantly worse for Aboriginal and American Indians in two studies [4, 28]. Inflammatory markers were found to be significantly higher in Aboriginal patients in one study [4], not significantly different between First Nations and Caucasians in another study [29], showing a trend toward being higher in American Indians [28], and improving at a slower rate during biologic therapy for Aboriginal patients in a third study [30].

#### Rheumatoid arthritis patient-reported outcomes

In comparison studies, most patient-reported outcomes including physical function measured by the Health Assessment Questionnaire, pain, patient global evaluation and fatigue were found to be worse in the Indigenous populations [4, 28–30] (Table 2). After 1 year of biologic treatment, EQ-5D, SF-36 MCS and SF-36 PCS were worse in Aboriginal compared to non-Aboriginal patients after adjustment for covariates [30]. However, quality of life using Cantril’s Global QOL index and a component-specific QOL instrument were assessed in one study, with no significant differences found between American Indians and Caucasians [27].

**Table 2:** Patient-reported outcomes studies in rheumatoid arthritis in Canadian, American, Australian and New Zealand Indigenous Populations

Patient-reported outcome	Study	Finding
Health Assessment Questionnaire (HAQ) score (or modified, mHAQ)	Peschken [4]	Mean scores at baseline similar between groups with disease duration <5 years and >15 years but higher in Indigenous groups at last visit (<5 years 0.90 Aboriginal vs 0.67 Caucasian; >15 years 1.21 Aboriginal vs 1.02 Caucasian) Worse scores at baseline (0.72 vs 0.56) and at last visit (1.19 vs 0.86) in Aboriginal for disease duration 5–15 years
	Poole [27]	HAQ score lower in American Indian vs Caucasian patients (1.0 vs 1.4)
	O’Neil [29]	No differences in mHAQ score at first visit but higher mHAQ scores at last visit in First Nations vs Caucasian patients (0.71 vs 0.42)
	Templin [31]	HAQ score in American Indian patients of 1.1 at time of study, 1.9 when asked as an ‘ever’ question
	Poole [44]	HAQ score of 1.8 in American Indian patients, increasing to 2.3 if comorbid diabetes
	Poole [47]	HAQ score of 1.0 in American Indian patients, increasing to 1.6 if comorbid diabetes
Pain (/100)	Genovese [28]	Higher pain score in American Indian vs Caucasian patients (64 vs 54)
	Peschken [4]	Higher pain scores in Aboriginal vs Caucasian patients at all lengths of disease duration (<5 years 50 vs 39; 5–15 years 48 vs 39, >15 years 51 vs 45)
	Barnabe [30]	Higher pain scores in Aboriginal vs non-Aboriginal patients at biologic start (76 vs 67)
Patient Global Score (/100)	Hitchon [26]	Worse score in First Nation patients with early disease and in late disease (43 vs 40)
	Peschken [4]	Worse scores in Aboriginal vs Caucasian patients at all lengths of disease duration (<5 years 45 vs 31; 5–15 years 40 vs 30, >15 years 40 vs 33)
	O’Neil [29]	No differences in score between First Nations and Caucasian patients at first visit
Fatigue (/100)	Peschken [4]	More fatigue in Aboriginal vs Caucasian patients at all lengths of disease duration (<5 years 55 vs 45; 5–15 years 50 vs 45, >15 years 53 vs 49)
AM stiffness	Genovese [28]	Same duration of morning stiffness between American Indian and Caucasian patients (median 60 min)
Global Quality of Life (QOL)	Poole [27]	No significant differences between American Indian and Caucasian patients at present, 5 years past or 5 years future
Component-specific Quality of Life	Poole [27]	No significant differences between Indigenous and Caucasian patients
EQ5D	Barnabe [30]	After 1 year of biologic treatment worse EQ-5D scores in Aboriginal vs non-Aboriginal patients (adjusted difference −0.07, 95% CI −0.11 to −0.03)
SF-36 Mental Health Composite Score	Barnabe [30]	After 1 year of biologic treatment worse SF-36 MCS in Aboriginal vs non-Aboriginal patients (adjusted difference −3.59, 95% CI −5.05 to −2.13)
SF-36 Physical Composite Score	Barnabe [30]	After 1 year of biologic treatment lower SF-36 PCS in Aboriginal vs non-Aboriginal patients (adjusted difference −2.34 (95% CI −3.90 to −0.78)

Global QOL: Cantril Self-Anchoring Scale (1965)

Component-specific QO: Dartmouth Primary Care Cooperative Information Project (COOP)

#### Rheumatoid arthritis radiographic findings

Studies describing the frequency of radiographic damage in rheumatoid arthritis were published prior to the advent of treat-to-target or biologic therapeutic strategies, limiting their relevance to current day practice; additionally, no studies had a comparison population. In the study of Pima Indians, 56% of subjects meeting 1987 ACR criteria and 100% of subject meeting 1961 Rome Criteria had erosive disease [41], and in a Chippewa Band, 36% had erosions and 55% typical radiographic changes for RA [37]. The Kiowa Indians all had characteristic changes on X-ray reported [40]. In a study of Tlingit Indians, 76% had erosions [31], whereas 90% of Alaskan Yupik Eskimos had characteristics changes [38], and 83% of a Canadian Inuit population had erosive changes [20]. Two studies reported on Kellgren and Lawrence stages of RA on radiographs in the Yakima Indian population; in an initial study by Beasley, 76% had Stage IV changes and 24% had Stage I changes [34]. This was followed by a larger study by Willkens where 64% had Stage IV changes compared to 13% of controls [35].

#### Rheumatoid arthritis serology

There were 20 studies that reported the frequency of RF positive (RF+) rheumatoid arthritis. In American Indians (*n* = 9 studies), the proportion of RF+ disease ranged from 13 to 100% [17, 31, 32, 34–37, 39, 40] while 78–87% of disease was RF+ in Alaska Natives (*n* = 2 studies) [38, 49]. In Canadian Aboriginals (*n* = 1 study), 89% were RF+ [4] while in Canadian First Nations groups (*n* = 7 studies) the frequency of RF+ ranged from 50 to 94% [33, 42, 43, 45, 46, 48, 50]. There was only one study in the Canadian Inuit population of which 83% were RF+ [20]. Of these 20 studies, only one compared the frequency of RF+ between Aboriginal and Caucasian populations, with a frequency of 89 and 74%, respectively [4]. The frequency of anti-CCP+ rheumatoid arthritis in American Indians was 55% in one study [17] while 5 studies in Canadian First Nations demonstrated the frequency ranging from 64 to 91% [45, 46, 48–50]. ANA+ frequency in rheumatoid arthritis cohorts ranged from 27 to 94% in 6 studies of American Indian populations [17, 31, 35–37, 40], 28% in an Alaska Native population [38], 57% in a Canadian Aboriginal cohort versus 21% in the Caucasian controls [4] and 75–77% in 3 studies in Canadian First Nations [42, 43, 45]. ANA was not detected in the Canadian Inuit population studied [20].

#### Rheumatoid arthritis summary

With a younger onset of disease, high rates of seropositive disease, and more frequent extra-articular features and comorbidities, Indigenous patients would be expected to encounter a more severe disease course, and less significant improvements in disease activity measures and patient-reported outcomes were indeed demonstrated in most studies. There is limited information on time to treatment and treatment strategy in the identified publications, which is critical information to include in future studies on the topic, especially with the advances experienced in the discipline of rheumatology over the past several decades.

### Juvenile idiopathic arthritis

Our review identified nine studies in Indigenous patients with juvenile idiopathic arthritis: six from Canadian First Nations [51–56], one from Canadian Inuit [20] and two in Alaska Native [57, 58] populations. In Canadian First Nations compared to Caucasian children, RF+ polyarticular juvenile idiopathic arthritis was more frequent (42 vs 3%), whereas pauciarticular disease was less frequent (22 vs 57%) [55]. Age of onset was not different between populations, but First Nations with RF+ polyarticular disease had a higher frequency of ANA (93 vs 44%) [55]. Onset subtype was not different for on vs off-reserve populations [55]. In Vancouver’s Children’s Arthritis Program in the 1960s, comparison between First Nations and non-First Nations children found no significant differences in clinical characteristics (fever, rash, iritis, pericarditis, poor growth, steroids, ankylosis or effusions); however, First Nations had more frequent RF+ disease (46 vs 5%) [53]. In a study of Toronto’s Hospital for Sick Children cohort in years 1984–2002, the onset type in First Nations varied from that of European populations, characterized as oligoarticular persistent (10 vs 30% European), oligoarticular extended (10 vs 12% European), RF-polyarticular (40 vs 23% European), RF+ polyarticular (20 vs 2% European) and systemic (10 vs 13% European), with no cases of psoriatic or enthesitis-related disease in First Nations but with these in 12 and 8% in European children, respectively [52]. First Nations patients had the highest rate of ANA+ arthritis, but their risk of uveitis was not elevated.

In a study with data for years 1976–1980, First Nations children with juvenile idiopathic arthritis from Vancouver and Winnipeg in Canada were compared to Caucasian children [51]. The First Nations children more frequently had a polyarticular onset subtype (59 vs 27%) and RF+ (36 vs 9%). Numerically but not statistically significant differences between First Nations and Caucasian children with juvenile idiopathic arthritis included a later onset age (8.5 vs 5.3 years), more joint involvement (mean 16 vs 9), less frequent pauciarticular disease (29 vs 61%), less risk of uveitis (12 vs 27%), more frequent ANA+ (53 vs 29%) and more frequent HLA-B27 (31 vs 15%) [51]. In further studies, First Nations race and residence on reserve were correlated with worse physical function scores and longer active disease duration in univariate analysis, and residing on reserve was predictive of worse disability in pauciarticular onset disease in multivariate analysis [54]. In a small Canadian Inuit population, polyarticular (*n* = 1) and pauciarticular disease (*n* = 3) was described [20]. A chart review was performed to characterize juvenile idiopathic arthritis subtype in a group of Inupiat children (*n* = 2 cases of seronegative enthesitis-related arthritis, *n* = 1 systemic onset, *n* = 2 reactive arthritis, *n* = 1 ankylosing spondylitis), with one third of these children having documentation of iritis [58]. In Alaska Natives, onset subtypes were described as follows: early onset (<7 years) pauciarticular ANA+ 21%, older onset pauciarticular or seronegative enthesitis-related arthritis 37%, RF+ polyarticular 32% and reactive arthritis 11% [57]. In the Yupik population, 4% had early onset pauciarticular ANA+ subtype, whereas 8% had early onset pauciarticular ANA-disease, with 71% having older onset pauciarticular or seronegative enthesitis-related arthritis, and 17% having reactive arthritis or ankylosing spondylitis [57]. In the Inupiat population, 20% had systemic disease, 33% had older onset pauciarticular or seronegative enthesitis-related arthritis, 33% had reactive arthritis, and 20% had ankylosing spondylitis [57]. There is a clear pattern of increased frequency of the polyarticular juvenile idiopathic arthritis subtype in Indigenous children of lower latitudes compared to other population groups, and a higher frequency of autoantibody positivity, yet with no increase in risk of eye complications; in contrast Indigenous children from northern populations have a predominant phenotype of pauciarticular and enthesitis-related arthritis, similar to findings in adult populations described later in this manuscript.

### Inflammatory arthritis

Seven studies describe either disease characteristics or disease activity in groups of Indigenous patients who did not meet classification criteria for a specific rheumatic disease at the time of study [16, 17, 37, 42, 59–61]. Canadian First Nations with inflammatory arthritis were younger and more likely to be seropositive, had higher DAS28-3ESR scores [59] and were less likely to be in remission after 12 months compared to non-First Nations (23 vs 48%, significant) [61]. In a study of descriptions of joint pain in American Indians with inflammatory joint disease (*n* = 12), the mean swollen joint count was 11, and the mean tender joint count was 18, with evidence of physical function impairment and high levels of pain (6.7 out of 10) described [16]. The remainder of studies described the frequency of serology findings in those with inflammatory arthritis not meeting criteria for specific rheumatic diseases. A cohort of western Canadian First Nations with episodic joint swelling were found to be frequently seropositive (35% RF+, 31% ANA+) and also has many features of connective tissue diseases [42]. A small cohort of Canadian Inuit with either a polyarticular or pauciarticular presentation were all seronegative for RF and ANA [20]. In the Oklahoma American Indian population with polyarthritis, 73% were ANA+ [17]. In the Chippewa American Indian bands with peripheral polyarthritis, 22% were RF+ and 33% were ANA+ [37]. It is interesting to consider that despite increased disease activity and the high frequency of autoantibody results, it was not possible to classify inflammatory arthritis more specifically, which may reflect patients presenting with ‘overlap’ type features and ultimately delay institution of appropriate therapy.

### Spondyloarthritis

Thirteen studies characterizing spondyloarthropathies in Indigenous populations were identified; one of these was on ankylosing spondylitis in three First Nations populations in Canada (Bella Bella, Bella Coola and Haida) and an American Indian population (Pima) in the USA [62], one was from a Canadian Inuit population with spondyloarthritis [20], two from Canadian First Nations populations with ankylosing spondylitis (Haida) [63] and spondyloarthritis (Nuu-Chah-Nulth) [42], two were from the Navajo population with ankylosing spondylitis and reactive arthritis in the USA [64, 65], and seven included analyses on a cohort of Alaskan Native patients with conditions including ankylosing spondylitis, reactive arthritis, psoriatic arthritis and undifferentiated spondyloarthritis [38, 66–71].

In the Navajo population, 80% of ankylosing spondylitis patients were HLA-B27 positive and 43% had knee involvement [64]. Seventy two percent with reactive arthritis had the characteristic triad of arthritis, conjunctivitis and urethritis, 88% were HLA-B27 positive, 53% had radiographic sacroiliitis, and 33% had uveitis or iritis [65]. The publication on the Nuu-Chah-Nulth population described patients with sacroiliitis and peripheral arthritis in the absence of extra-articular findings and without confirmation of ankylosing spondylitis, as well as one case of reactive arthritis with peripheral arthritis and urethritis features [42]. In 10 males with ankylosing spondylitis from the Haida population, 90% had Grade 2–3 radiographic changes by the Carter scale, 80% had a history of peripheral joint symptoms, and 30% were confirmed to have iritis, whereas another 20% had a history of eye inflammation [63]. Canadian Inuit with either spondyloarthropathies were characterized for clinical features; the majority had mild functional class and disease activity limitations [20].

In the Alaska Native population, 5-year follow-up on the original set of cases is described [69]. All ankylosing spondylitis patients had Grade 2–4 sacroiliitis on radiography compared to only 33% of undifferentiated spondyloarthritis and 57% of reactive arthritis patients. When combining all cases of spondyloarthritis, over two thirds had loss of spinal motion, 42% had limited chest expansion, and 78% had peripheral inflammatory arthritis, with the knee being most commonly involved. Iritis or uveitis affected 13% of spondyloarthritis patients (36% of those with ankylosing spondylitis). In this publication, patients were also assigned to a severity category of disease (mild, moderate or severe), with modified HAQ scores varying from 0.2 to 0.9, the Dougados Functional Index Scores ranging from 1.8 to 8.4, and physician global scores ranging from 1.8 to 4.8 across severity categories, although the timing of assessment was not specified. This longitudinal study is helpful to confirm the severe impact of spondyloarthritis conditions in the Alaska Native population, as many of the studies were descriptive in nature and without comparison populations, limiting interpretations that can be drawn from the research.

### Juvenile spondyloarthritis

In a Canadian Inuit population, approximately half of those with juvenile-onset spondyloarthritis who had radiographs had Grade IV changes [20]. From another Canadian study, 103 patients with seronegative juvenile spondyloarthritis were identified; First Nations patients represented 9% of psoriatic arthritis, 19% of seronegative enthesitis and arthritis, 44% of ankylosing spondylitis and 67% of reactive arthritis cases, with no cases of inflammatory bowel disease-related spondyloarthritis [56]. In a study from 1976 to 1980, First Nations children with spondyloarthritis from Vancouver and Winnipeg were compared to Caucasian children [51]. Onset age and the frequency of HLA-B27 positivity were similar between populations (70 vs 64%), and joint involvement was slightly higher in First Nations children but not significantly (mean 6.6 vs 4.6); however, First Nations children had more frequent eye inflammation (36 vs 4%) [51].

### Crystal arthritis

A single study comparing demographic and disease features of gout in 342 New Zealand Maori and 315 Europeans was identified [72]. Maori were younger at onset (46 vs 50 years), with 90% of those with a polyarticular course being Maori, and tophi in 1.2% of Maori and 0.3% of Europeans.

### Systemic lupus erythematosus

There were 19 systemic lupus erythematosus studies identified [5, 17, 42, 57, 73–87] with data for Indigenous populations in all four countries available. Age at onset of disease was similar in Indigenous and Caucasian groups in both Canada [73] and Australia [87]. In the Oklahoma American Indian population, the average number of ACR classification criteria met was 5.3 (range 4–7) [17]. In an Australian Aborigine population, the mean number of ACR criteria met was 5 (range 4–7) compared to 7 (range 5–8) in the Caucasian comparison cohort [86]. Puar did not specify the number of criteria met, but noted there was no difference between the Canadian First Nations and Caucasian populations [77]. In the 1000 Canadian Faces of Lupus study, the mean number of ACR criteria met was 5.7 (SD 1.7) in Aboriginals, and 6.0 (1.7) in Caucasians, however, not significantly different [73].

#### Systemic lupus erythematosus non-renal manifestations (Table 3)

There was a wide variation in the frequency of non-renal classification criteria manifestations between individual studies in different Indigenous populations. One Canadian study highlighted more frequent arthritis in First Nations compared to Caucasian patients [77], but otherwise there did not appear to be any significant differences in that manifestation in either Canadian Aboriginal or First Nations populations compared to Caucasian controls. There were no comparison studies of non-renal manifestations in the American literature between the Indigenous and Caucasian population. In the Australian Aboriginal population, Segasothy reported less frequent photosensitivity, malar rash, discoid rash, oral ulcers, serositis in Indigenous patients but more frequent hematologic findings [86]. Bossingham reported less frequent photosensitivity [87].

**Table 3:** Systemic lupus erythematosus organ manifestations in Canadian, American, Australian and New Zealand Indigenous Populations Compared to the Caucasian Population (where available)^a^

Criteria	Aboriginal, Canada	First Nations, Canada	American Indian	Alaska Native	American Indian/Alaska Native	Australian Aborigine	New Zealand Maori
Photosensitivity	41 vs 57% [73]	75% [42] 39 vs 42% [5]	69% [17]	39% [57]	53%^#^ [85]	14% [82] 11 vs 50%^b^ [86] 11 vs 39%^b^ [87]	NR
Malar rash	54 vs 64% [73]	12.5% [42] 56 vs 50% [5]	69% [17]	46% [57]	32% [85]	27% [82] 44 vs 83%^b^ [86]	NR
Discoid rash	13 vs 20% [73]	24 vs 25% [5]	0% [17]	15% [57]	8%^#^ [85]	9% [82] 28 vs 67%^b^ [86]	NR
Oral ulcers	49 vs 58% [73]	38% [42] 17 vs 33% [5]	32% [17]	15% [57]	35%^#^ [85]	14% [82] 17 vs 50%^b^ [86]	NR
Serositis	37 vs 32% [73]	50% [42] 33 vs 27% [76] Pericarditis 22 vs 12% [5] Pleuritis 25 vs 26% [5]	38% [17]	62% [57]	48% [85]	Pericarditis 32% [82] 22 vs 17% [86] Pleuritis 45% [82] 50 vs 83%^b^ [86]	NR
Arthritis	74 vs 82% [73]	90 vs 82% [5] 90 vs 67%^b^ [77]	88% [17]	92% [57]	80%^#^ [85]	64% [82] 78 vs 83% [86] 76 vs 90% [87]	NR
Neurologic	14 vs 6% [75]	13% [42] 13 vs 9% [76] Seizures 10 vs 3% [5] Psychosis 10 vs 6% [5]	32% [17]	31% [57]	3% [85]	5% [82] 6 vs 17% [86] 34 vs 48% [87]	NR
Hematologic	60 vs 70% [73]	Leukopenia 70% [42] 33 vs 43% [5] Thrombocytopenia 29 vs 15% [5] Hemolytic anemia 13 vs 4% [5] Lymphopenia 60 vs 65% [5]	44% [17]	54% [57]	90% [85]	Leukopenia 5% [82] 44 vs 33% [86] Thrombocytopenia 32% [82] 39 vs 17%^b^ [86] Hemolytic anemia 9% [82] 14 vs 0% [86] Lymphopenia 64% [82] 77 vs 33%^b^ [86] Anemia 38 vs 31% [87]	NR
Renal	*Ever* Renal disease 39 vs 40% [73]	*At diagnosis* Proteinuria 22 vs 16% [5] Casts 17 vs 6%^b^ [5] *Ever* Proteinuria 46 vs 25%^a^ [5] Casts 35 vs 12%^b^ [5] Nephritis 48 vs 29% [77] 57 vs 32%^b^ [76] Renal disease 0% [42]	*At diagnosis* Nephritis 21 vs 12% [81] *Ever* Renal disease 32% [17]	*Ever* Renal disease 39% [57]	*Ever* 40% [82]	*Ever* Proteinuria 22 vs 17% [86] 62% [82] Casts 11 vs 0% [86] 41% [82] Renal disease 46 vs 28% [87]	*At diagnosis* Nephritis 10 vs 4%^b^ [79] *Ever* Nephritis 17 vs 19% [79]^c^
ANA positive	96 vs 95% [73]	88% [42] 100 vs 98% [5]	69% [17]	100% [57]	98% [82]	100% [82] 100 vs 100% [86]	NR
Immunologic criteria	89 vs 83% [73]		38% [17]	77% [57]	61%^#^ [82]		NR

^#^Aggregate data presented. Publication provides combined rates and separated by region (Alaska, Phoenix, Oklahoma)—only arthritis, immunologic disorder, oral ulcers and discoid rash differ significantly between groups—with arthritis and discoid rash being more frequent in Phoenix, Immunologic disorder more frequent in Alaska, photosensitivity and oral ulcers being less common in Oklahoma

^a^Indigenous population % versus comparison population % if available; in all studies, the comparison population was Caucasian except for Bossingham [87] where the comparison was to the non-Indigenous Population

^b^Statistically significant difference

^c^After adjustment significant difference between groups [OR 8.47 (95% CI 2.11–33.96) vs all patients]

#### Systemic lupus erythematosus renal manifestations (Table 3)

Multiple studies have examined renal manifestations of SLE either at diagnosis, or during the disease course. Indigenous patients in Canada had significantly more renal casts but not proteinuria at diagnosis [5], whereas nephritis was more frequent in American Indian [81] and New Zealand Maori [79] populations. During the disease course, between 22 and 62% of the Australian Indigenous population were characterized as having proteinuria [82, 86] and 11–41% having cellular casts, which were features also significantly more frequent in Canadian Indigenous [5] populations compared to Caucasian controls. All three studies specifically assessing nephritis found it to be more frequent in the Indigenous populations, ranging from 17 to 57% in Indigenous versus 19–32% in controls [76, 77, 79]. Six studies described the frequency of ‘renal disease’ without further specification; with the exception of one outlier study [42], the estimated frequency of renal disease clustered between 32 and 46% [17, 57, 73, 85, 87].

#### Systemic lupus erythematosus serology

The frequency of positive anti-dsDNA varied from 20% [42] to 76% [5] in First Nations and was 68% [73] in the Canadian Aboriginal group. In the Australian populations, anti-dsDNA positivity varied from 42% [87], 56% [86] to 77% [82]. In none of these studies was anti-dsDNA more frequent in the Indigenous populations. In contrast, just 13% of the American Indian population studied had a positive anti-dsDNA [17]. All of Peschken [5], Hitchon [76] and Segasothy’s [86] studies identified more frequent anti-Sm and anti-RNP antibodies in the Indigenous populations. Segasothy additionally identified less frequent anti-cardiolipin antibodies and lupus anticoagulant in the Australian Aborigine population [86].

#### Systemic lupus erythematosus disease activity/damage

Four publications, two from the 1000 Canadian Faces of Lupus [73, 74] and two from the Manitoba SLE Cohort [5, 76], investigated differences in disease activity and damage between Indigenous and Caucasian populations. In the 1000 Canadian Faces of Lupus study, Aboriginal participants did not have significantly worse SLEDAI-2K scores, but a larger proportion were in the highest quartile of scores (35 vs 23%) and a lower proportion were in the lowest quartile (12 vs 31%) compared to Caucasians [73]. In the Manitoba cohort, First Nations participants had a higher mean SLEDAI at diagnosis but without significant differences in scores at 2 years or at last follow-up compared to Caucasians; however, they had more damage by the SLICC/ACR Damage Index at both follow-ups in both crude and adjusted analyses [5].

#### Systemic lupus erythematosus summary

Surprisingly, despite more frequent nephritis involvement and worse damage accrual, disease activity does not appear to be worse in Indigenous populations with lupus and there is no indication of a predominant non-renal phenotype or higher frequency of autoantibodies consistent across the populations studied. As in rheumatoid arthritis, lupus studies have not made much mention of treatment strategy, and this will be important to collect in the cohorts that have been established.

### Juvenile systemic lupus erythematosus

Seven studies in juvenile systemic lupus erythematosus were identified [88–94]. In a cross-sectional study of four Canadian pediatric rheumatology centers, Aboriginal patients had longer disease duration (4.3 vs 2.3 years) than other ethnicities, despite similar mean age at study [90]. Compared to White children, Aboriginal children with juvenile systemic lupus erythematosus had a significantly lower frequency of malar rash (33 vs 78%) and more frequent serositis (44 vs 11%), with no significant differences in the frequency of autoantibodies [90]. Disease activity indices (e.g., SLEDAI-2K, SLAM-R), a damage index (SDI), physician global evaluation and fatigue scores were similar across ethnicities, and health-related quality of life was not demonstrated to be significantly different among specific ethnicity groups but with limitations of unbalanced and small sample sizes [90, 94]. In the 1000 Canadian Faces of Lupus cohort, Aboriginal children with juvenile systemic lupus erythematosus had significantly elevated odds of developing serositis (OR 18.5, 95% CI 1.8–188.6) in multivariate analysis [91]. In a retrospective review of 22 First Nations children with juvenile systemic lupus erythematosus attending a single center in Vancouver, all five had lung involvement [93]. A publication in 2006 from this same center reported significantly higher frequency of manifestations of non-erosive arthritis (100 vs 32%), myositis (33 vs 0%), gastrointestinal symptoms (93 vs 9%) and the autoantibody anti-SSA (100 vs 53%) in First Nations compared to non-First Nations children with juvenile systemic lupus erythematosus, but with disease activity at presentation and damage at 6 months not being significantly different [89]. In an analysis of Medicaid enrollees from the USA, American Indians with juvenile systemic lupus erythematosus had a higher frequency of lupus nephritis, characterized by an incident rate of 1.61 (95% CI 0.72–3.58), compared to 0.30 (95% CI 0.21–0.43) in Whites [92]. Finally, in a cohort of New Zealand Maori and European children with systemic lupus erythematosus (years 2000–2010), there was no significant difference in age at diagnosis across ethnic groups [88]. In this small sample, there were statistically significant differences in disease phenotype, with serositis affecting 50% of Maori compared to 10% of European children, lupus nephritis affecting 75% of Maori versus 40% of European children with a higher frequency of World Health Organization class 4 or 5 lesions (50 vs 40%, respectively, although with no significant differences in disease activity [88]. In juvenile systemic lupus erythematosus, differences in phenotype do seem to exist, and longitudinal outcomes will be important to examine to understand the impact of these phenotypes on damage accrual and mortality.

### Scleroderma

Three studies were identified describing scleroderma clinical features in Canadian First Nations and American Indian populations [95–97] (Table 4). The American Indian studies involved the same population of patients of Choctaw descent; Arnett’s study reported on 17 subjects, but did not involve a comparison to another population [96], whereas Kuwana’s study included 12 of these subjects but in comparison with Caucasian patients [97]. The mean age of disease onset was 4 years younger in Canadian First Nations compared to the non-First Nations population [95]. In Choctaw Native Americans, age at disease onset was also younger compared to the Caucasian population (53 vs 42 years) but this was not statistically different [97]. There were no significant differences in the cutaneous subtype (limited vs diffuse disease) nor mean Rodnan skin scores between Canadian First Nations and Caucasian patients [95]; the majority of the Choctaw patients had diffuse disease, not different in frequency to the Caucasian population [97]. Polyarthritis was more frequent in the Canadian First Nations population [95], but not the Choctaw American Indian population [97] relative to the Caucasian populations. Canadian First Nations had a higher mean number of gastrointestinal symptoms reported, at a mean of 5.8 versus 4.1 in the Caucasian population, as well as worse gastrointestinal symptom severity (mean 2.9 vs 1.7 on 0–10 scale) [95]. Raynaud’s phenomenon severity (mean 3.9 vs 2.8 on a 0–10 scale) was also worse in the Canadian First Nations population [95]. All studies described above as well as a study by Gaddy [17] examined the frequency of autoantibodies in patients with scleroderma, with a wide range of variation in the frequency of their presence, but with no significant differences found between Indigenous populations and Caucasian comparison groups. Thus, the available literature highlights variations in scleroderma phenotype in Canadian First Nations populations compared to Caucasians, whereas phenotype was not different in the American setting.

**Table 4:** Scleroderma features in Canadian and American Indigenous populations

	First Nations, Canada (*n* = 71) vs Caucasian (*n* = 1038) [95]	American Indian Choctaw (*n* = 17) [96], vs Caucasian (*n* = 12 vs *n* = 47) [97]
Diffuse skin involvement	46.5 vs 35.6%	64.7% 75.0 vs 66.0%
Finger contractures	NR	67.0 vs 72.0%
Telangiectasias	NR	92.0 vs 72.3%
Lung fibrosis	29.6 vs 33.7%	88.2% 92.0 vs 57.0%^a^
Pulmonary hypertension	8.5 vs 11.0%	NR
Raynaud’s phenomenon	NR	88.2% 92.0 vs 94.0%
Digital ulcers	63.4 vs 52.1%	NR
Polyarthritis	44.8 vs 30.5%^a^	83.0 vs 74.0%
Myositis	12.7 vs 10.4%	NR
Scleroderma renal crisis	4.3 vs 3.9%	NR
Renal	NR	0 vs 4%
Overlap with other disease	24.3 vs 14.9%^a^	NR
Gastrointestinal symptoms (mean, SD)	5.8 (3.2)	4.1 (3.1)

*NR* not reported

^a^Statistically significant difference

### Sjogren’s

A single study reporting on serology in patients with rheumatic diseases from the Oklahoma American Indian population included a single patient with Sjogren’s syndrome, who was positive for ANA, Anti-Ro and RF antibodies [17].

## Discussion

We have assembled the available descriptions of rheumatic disease clinical features in Indigenous populations of Canada, America, Australia and New Zealand. The purpose of the work was to advance beyond descriptions of disease prevalence alone as a reflection of arthritis burden in Indigenous communities and summarize the literature on disease characteristics, severity and outcomes. In rheumatoid arthritis, measures of disease activity and all studies describing patient-reported outcome measures of pain, function, patient global evaluation, fatigue, quality of life and well-being indicate a more negative impact of this disease in Indigenous populations in North America. Juvenile idiopathic arthritis in North American Indigenous populations is characterized by a higher frequency of polyarticular disease subtype. Disease manifestations in Indigenous populations with systemic lupus erythematosus vary from that of general populations in the respective countries studied; arthritis and renal disease were more frequent in the Canadian First Nations populations, with less frequent cutaneous manifestations and serositis in Australian Aborigine populations. In juvenile systemic lupus erythematosus, both American Indian and New Zealand Maori populations experience nephritis at a greater frequency, and serositis is more frequent in both Canadian Aboriginal and New Zealand groups, than in the general population. In particular, Canadian Aboriginal children with systemic lupus erythematosus are more likely to have lung involvement, arthritis, myositis and gastrointestinal manifestations. Scleroderma phenotype in Canadian First Nations people is characterized by more frequent polyarthritis, gastrointestinal symptoms and Raynaud’s phenomenon compared to Caucasians, whereas phenotype was not different between American Indian and Caucasian populations. The lone study found that described gout phenotype was from New Zealand, with Maori people more frequently having polyarticular disease and a higher frequency of tophi compared to the European population. In the Maori population with osteoarthritis undergoing joint replacement surgery, worse preoperative function, postoperative functional improvements and mental health scores were evident. In the American Indian/Alaska Native population with self-reported arthritis, more activity limitations, worse physical scores and a higher impact on work were identified compared to the general population.

Our results highlight gaps in the current knowledge base; most studies focus on singular rheumatic diseases in select populations in North America, with few studies from Australia and New Zealand. Many studies were performed prior to the significant advances in early diagnosis and targeted management strategies in rheumatology, which would be expected to provide beneficial impacts on outcomes. The spondyloarthropathy literature identified in our review does not allow for comparisons to the general population, and primarily described disease phenotype in populations, rather than the impact of disease on function, quality of life and well-being. Few studies were longitudinal in design, limiting assessment of outcomes as was one of our original goals.

Beyond being a summary document, this review enables us to consider aspects of observational research in rheumatology pertaining to Indigenous populations. The literature perpetuates classic western biomedical model perspectives on outcomes, without considering if these outcomes are indeed relevant to Indigenous populations, or if they appropriately consider patient roles in a community context rather than the individualistic focus. This literature is remarkable in its lack of a health equity lens on outcomes and does not delve into the fact that some phenotypic and outcome differences seen may be explainable by avoidable causes. These points are critical in informing how we proceed to deconstruct practices that reinforce health inequities and proceed with establishing effective models of care with appropriate evaluation frameworks. The opportunity now is to harness and leverage the evidence to advocate for in-depth study, ensuring that principles of community-based participation and ownership of research are upheld, thereby creating an opportunity to deliver on promises made in the signing of treaties for effective care.

This work builds on the existing reviews related to the epidemiology of rheumatic disease in Indigenous populations of North America (1), and rheumatoid arthritis epidemiology and clinical features in the American Indian and Alaska Native populations (2), by updating the literature searches and expanding the scope to include Australian and New Zealand populations; epidemiology updates and an evaluation of health services utilization are published [3, 98]. Ideally, our review would have allowed meta-analysis; however, population heterogeneity and insufficient data precluded this activity. In the interest of conciseness, we grouped Indigenous populations within countries in our summaries; the heterogeneity in Indigenous populations within countries is not to be forgotten. In our search and article selection, we endeavored to eliminate studies reporting duplicate and/or overlapping data, a concern with situations where multiple studies from the same population in overlapping years were identified, but cannot verify that patients belonged uniquely to each study, and rather suspect there may be instances where patients contributed data to several studies. Publication bias may favor us locating studies where differences between populations are found. Finally, we recognize that Indigenous populations in other countries likely also face rheumatic disease inequities that require further understanding and action. For example, the work of GLADERPO (Grupo Latino Americano de studio De Enfermedades Reumaticas en Pueblos Originarios) carries out epidemiological, genetic and anthropological studies related to rheumatic diseases in Indigenous peoples of Latin America [99].

## Conclusions

The existing literature supports differences in disease phenotype and severity in Indigenous populations of Canada, America, Australia and New Zealand. We encourage investigators in this area of research to undertake contemporary studies that disentangle differences between phenotype and severity that merely reflect differences in access to care and that provide a longitudinal view of outcomes in more diverse populations. These findings would be instrumental to informing health service planning that resolves health inequities.

## Appendix: MEDLINE search

**Table:**

Rheumatoid arthritis	1.	rheumatoid arthritis.tw. or exp arthritis, rheumatoid/
	2.	((rheumatoid or reumatoid or revmatoid or rheumatic or reumatic or revmatic or rheumat* or reumat* or revmarthrit*) adj3 (arthrit* or artrit* or diseas* or condition* or nodule*)).tw.
	3.	(felty* adj2 syndrome).tw.
	4.	(caplan* adj2 syndrome).tw.
	5.	(sjogren* adj2 syndrome).tw.
	6.	(sicca adj2 syndrome).tw.
	7.	still* disease.tw.
	8.	1 or 2 or 3 or 4 or 5 or 6 or 7
Ankylosing Spondylitis (AS) and other spondyloarthropathies (include psoriatic arthritis and Reiter’s disease.)	9.	exp Spondylitis, Ankylosing/
	10.	(ankylos* or spondyl*).tw.
	11.	(bekhterev* or bechterew*).tw.
	12.	(Marie adj struempell*).tw.
	13	exp Arthritis, Psoriatic/
	14	(psoria* adj (arthriti* or arthropath*)).tw.
	15	((arthriti* or arthropath*) adj psoria*).tw.
	16	exp Spondylarthropathies/
	17	exp Arthritis, Infectious/
	18	reactive arthritis.tw.
	19	(reiter* adj (disease or syndrome)).tw.
	20	((sexual* or chlamydia or yersinia or postyersinia or postdysenteric or salmonella or shigella or b27 or postinfectious or post infectious) adj5 arthrit*).tw.
	21	reactive enthesitis.tw.
	22	undifferentiated oligoarthritis.tw.
	23	9 or 10 or 11 or 12 or 13 or 14 or 15 or 16 or 17 or 18 or 19 or 20 or 21 or 22
Osteoarthritis	24	exp Osteoarthritis/
	25	osteoarthr*.tw.
	26	(degenerative adj2 arthritis).tw.
	27	24 or 25 or 26
Gout	28	exp Gout/
	29	gout*.tw.
	30	(tophus or tophi or tophaceous).tw.
	31	28 or 29 or 30
Connective tissue disorders include systemic lupus erythematosus, scleroderma, Connective Tissue disorders: polymyositis, dermatomyositis, and Sjögren’s syndrome	32	exp connective tissue diseases/
		connective tissue disease*.tw.
	33	exp lupus erythematosus, systemic/
	34	(SLE or lupus).tw.
	35	exp Scleroderma, Systemic/or exp Scleroderma, Localized/
	36	scleroderma.tw.
	37	(systemic adj3 sclerosis).tw.
	38	exp Polymyositis/
	39	polymysositis.tw.
	40	exp Dermatomyositis/
	41	dermatomyositis.tw.
	42	exp Sjogren’s Syndrome/
	43	(sjogren* adj2 syndrome).tw
	44	32 or 33 or 34 or 35 or 36 or 37 or 38 or 39 or 40 or 41 or 42 or 43 or 44
Idiopathic juvenile arthritis	45	exp Arthritis, Juvenile/
	46	idiopathic juvenile arthritis.tw.
	47	46 or 47
General arthritis and rheumatic disease terms	48	exp arthritis/
	49	exp rheumatic disease/
	50	(arthrit* or rheum*).tw.
	51	49 or 50 or 51
All Arthritis	52	8 or 23 or 27 or 31 or 45 or 52
Indigenous: International	53	(aborig* or indig* or tribe or tribes or tribal or natives or native people or first people or peoples).tw.
	54	exp Health Services, Indigenous/
	55	54 or 55
Indigenous: Canada/United States specific	56	(inuit* or eskimo* or metis or indian or indians or Amerindian* or (Native adj3 (America* or Alaska* or Canad*)) or (First adj (nation* or Canad* or America*))).tw.
	57	exp United States Indian Health Service/or American Native Continental Ancestry Group/or exp Indians, North American/or exp Inuit/
	58	57 or 58
Indigenous Australia/New Zealand specific	59	(Maori* or Torres Strait islander* or (Pacific adj (Islander* or People*)) or First Australian*).tw.
	60	exp Oceanic Ancestry Group/
	61	60 or 61
All indigenous	62	56 or 59 or 62
Combo	63	53 and 63
Remove animals	64	limit 64 to animals
	65	limit 65 to (animals and humans)
	66	65 not 66
	67	64 not 67
